# Editorial: Recent advances in research and development for vegetable crops under protected cultivation

**DOI:** 10.3389/fpls.2024.1459919

**Published:** 2024-07-24

**Authors:** Giao N. Nguyen, Zora Singh

**Affiliations:** ^1^ Department of Primary Industries and Regional Development, Carnarvon, WA, Australia; ^2^ Horticulture, School of Science, Edith Cowan University, Joondalup, WA, Australia

**Keywords:** protected cropping, vegetable, hydroponics and soilless culture, substrate, greenhouse lighting control, root zone temperature, biochar, peat

Protected cultivation is the production of horticultural crops including vegetables under structures such as forced-ventilated greenhouses, shade net houses, poly houses, net houses, high and low plastic tunnels, where the external growing environment can be controlled or modified to suit crop growth requirements. In regions where the environmental conditions such as weather, soil and water are not suitable to grow specific vegetables, or in urban areas with limited arable land, this farming system is very important to ensure high, stable productivity and good quality vegetables. This enables a stable supply for the market all year round, bringing high economic return to growers. This research area has been receiving much attention from the commercial vegetable production sector and scientific community. With the aim to contribute to the understanding of this research area, this Research Topic was designed to collect the latest scientific research and development in protected cropping for commercial vegetable production. Eight original research and review articles were already published with the invaluable contribution of 54 authors coming from 20 research institutions in 7 countries. This editorial summarises key highlights from the articles contributed to this theme.

To gain an insight into current vegetable production systems, Ahmed et al. provided a comprehensive review on various aspects of modern vegetable production. The authors analysed and discussed the transition from traditional cultivation to modern greenhouse vegetable production methods. The review emphasised the role of scientific research and advanced production support tools for precise fertigation, irrigation and integrated pest management. The utilisation of drones, robots and digital monitoring systems for vegetable production was also intensively discussed.

Having suitable, affordable and environmentally friendly growing media substrates for specific vegetable crops for high yield and good quality is critical for a successful soilless growing method under protected cultivation. Although peatmoss is widely used for as an ideal substrate for hydroponic vegetables, it raises a copious number of environmental concerns. To this purpose, Yu et al. dedicated their work to review the use of biochar as an alternative subtract to peatmoss. They suggested that biochar can be used for greenhouse and nursery production and brings environmental and economic benefits. Under their view, biochar is a source of renewable materials that can be created from many different materials available in nature. Further on this subject, Adamczewska-Sowin´ska et al., reported on a more suitable material, willow tree chips which are environmentally friendly and abundant in nature, as an alternative substrate for growing cucumbers. The authors evaluated a total of 29 mixtures using potting materials such as willow, peat, biochar, and basalt meal and found that willow chips could be used as a partial substitute for environmentally harmful peat.

Modifying unfavourable environmental conditions such as light, temperature and humidity to facilitate crop growth is critical for vegetable production under protected cropping. In this Research Topic, four papers provided an insight the role of light and its interaction with environmental factors to cause an impact on crop metabolism, yield and quality under this theme. Using appropriate materials to build a greenhouse is extremely important, ensuring light and temperature for plant photosynthesis, while saving energy to reduce costs and increase the competitiveness of fresh produce. Maier et al. conducted a thorough investigation on the effects of light blocking film to the growth, development and yield of capsicum under various seasonal conditions. Their research findings proved that the choice of right roof cover when constructing a greenhouse is essential to ensure that adequate light quantity and quality can be achieved for optimum crop growth. Likewise, Ramezani et al. highlighted the role of different light spectra generated by different light-emitting-diodes and nitrogen supplementation on spinach in a controlled environment agriculture platforms. The authors provided an insight into the interaction between light spectra combinations with an emphasis on green light and nitrogen modulating the spinach yield and quality. These results again confirmed the importance of light source selection for protected cropping. The importance of the supplementary light source was again reported by an interesting study of Moratiel et al., where the interplay between light spectra, their intensities and carbon dioxide concentrations on the net photosynthesis of tomatoes seedlings was thoroughly investigated. The results from this study showed that the properties of the spectrum are essentially crucial under low light conditions to achieve the optimum crop’s net photosynthesis. The study of Begum et al. on chilling resistant sweet basil further emphasized on the importance of the light components. The authors demonstrated that a short duration of far-red light supplement four days before harvesting would alter several biochemical pathways and enhance the cold tolerance of sweet basil. These findings could be useful for a large-scale commercial basil production to reduce chilling injuries and improving self-life of fresh basil.

Maintaining optimal root zone temperatures are critical for the nutrient uptake of vegetable cultivation using soilless method since fluctuations in nutrient solution temperatures will cause changes in other attributes such as electrical conductivity and pH, which affect nutrient absorption at the root zone. Developing a cost-effective and energy efficient method that can maintain nutrient solution temperatures is essential. Nisar et al. designed and compared of the efficacy of four energy saving (non-electric) cooling methods for several vegetables in open-air hydroponics. The results showed that the cooling setup III outperformed the other three methods, and it could reduce the nutrient solution temperature up to 193%. Intuitively this method can potentially be applied for hydroponic systems in protected cropping to stabilize root zone temperatures while saving energy consumption which is usually a major cost for commercial vegetable production.

We hope that eight articles published in this Research Topic will provide the readers with up-to-date content related to vegetable production under protected cultivation. Nevertheless, further studies on greenhouse construction materials, and optimal designs suitable for various ecozones, that save energy and reduce production costs would be worth investigating. The selection of suitable crop varieties for protected cultivation for optimal yield and premium quality together with an integrated pest management system, using minimal pesticides, extending postharvest life and minimising postharvest losses also needs more attention from the vegetable scientific community. Furthermore, advanced agronomy methods such as using closed hydroponics system where the run to wastewater can be efficiently recycled to optimise water and fertilizer use could be interesting Research Topics in near future.

## Author contributions

GN: Writing – original draft, Writing – review & editing. ZS: Writing – review & editing.

